# Debate and Dilemmas Regarding Generative AI in Mental Health Care: Scoping Review

**DOI:** 10.2196/53672

**Published:** 2024-08-12

**Authors:** Xuechang Xian, Angela Chang, Yu-Tao Xiang, Matthew Tingchi Liu

**Affiliations:** 1 Department of Communication Faculty of Social Sciences University of Macau Macau SAR China; 2 Department of Publicity Zhaoqing University Zhaoqing City China; 3 Institute of Communication and Health Lugano University Lugano Switzerland; 4 Department of Public Health and Medicinal Administration Faculty of Health Sciences University of Macau Macau SAR China; 5 Faculty of Business Administration University of Macau Macau SAR China

**Keywords:** generative artificial intelligence, GAI, ChatGPT, mental health, scoping review, artificial intelligence, depression, anxiety, generative adversarial network, GAN, variational autoencoder, VAE

## Abstract

**Background:**

Mental disorders have ranked among the top 10 prevalent causes of burden on a global scale. Generative artificial intelligence (GAI) has emerged as a promising and innovative technological advancement that has significant potential in the field of mental health care. Nevertheless, there is a scarcity of research dedicated to examining and understanding the application landscape of GAI within this domain.

**Objective:**

This review aims to inform the current state of GAI knowledge and identify its key uses in the mental health domain by consolidating relevant literature.

**Methods:**

Records were searched within 8 reputable sources including Web of Science, PubMed, IEEE Xplore, medRxiv, bioRxiv, Google Scholar, CNKI and Wanfang databases between 2013 and 2023. Our focus was on original, empirical research with either English or Chinese publications that use GAI technologies to benefit mental health. For an exhaustive search, we also checked the studies cited by relevant literature. Two reviewers were responsible for the data selection process, and all the extracted data were synthesized and summarized for brief and in-depth analyses depending on the GAI approaches used (traditional retrieval and rule-based techniques vs advanced GAI techniques).

**Results:**

In this review of 144 articles, 44 (30.6%) met the inclusion criteria for detailed analysis. Six key uses of advanced GAI emerged: mental disorder detection, counseling support, therapeutic application, clinical training, clinical decision-making support, and goal-driven optimization. Advanced GAI systems have been mainly focused on therapeutic applications (n=19, 43%) and counseling support (n=13, 30%), with clinical training being the least common. Most studies (n=28, 64%) focused broadly on mental health, while specific conditions such as anxiety (n=1, 2%), bipolar disorder (n=2, 5%), eating disorders (n=1, 2%), posttraumatic stress disorder (n=2, 5%), and schizophrenia (n=1, 2%) received limited attention. Despite prevalent use, the efficacy of ChatGPT in the detection of mental disorders remains insufficient. In addition, 100 articles on traditional GAI approaches were found, indicating diverse areas where advanced GAI could enhance mental health care.

**Conclusions:**

This study provides a comprehensive overview of the use of GAI in mental health care, which serves as a valuable guide for future research, practical applications, and policy development in this domain. While GAI demonstrates promise in augmenting mental health care services, its inherent limitations emphasize its role as a supplementary tool rather than a replacement for trained mental health providers. A conscientious and ethical integration of GAI techniques is necessary, ensuring a balanced approach that maximizes benefits while mitigating potential challenges in mental health care practices.

## Introduction

### Background

Mental disease is among the top 10 leading causes of global burden [1]. One out of every 2 people worldwide will develop at least 1 mental disease in their lifetime [2]. Depression and anxiety disorders are among the most prevalent mental health conditions, significantly impacting individuals’ quality of life. It is estimated that approximately 280 million people worldwide are living with depression, with another 301 million experiencing anxiety [3]. These mental diseases can lead to debilitating symptoms, impairing social functioning, and affecting overall well-being [4,5]. People with depression or anxiety are also at a high risk of suicide. More than 700,000 people take their lives every year, with >77% of global suicides occurring in low- and middle-income countries [6].

Despite concerted efforts to address mental health problems, several challenges persist. Limited access to mental health services, especially in resource-limited countries, resulted in a huge treatment gap in mental health care [7]. The lack of sufficient trained mental health professionals further exacerbates this problem, leading to long waiting times for consultations and inadequate support [8]. In addition, stigma and discrimination surrounding mental health continue to hinder individuals from seeking the help they need. Many individuals feel ashamed or worried about the potential consequences of disclosing their mental health conditions, thus impeding early intervention and treatment [7].

Artificial intelligence (AI) appears as a viable alternative for facilitating accessibility, affordability, and anonymity in psychiatric diagnosis and treatment due to its capability to mimic human cognitive functions to learn and make decisions [9]. This is particularly true in machine learning (ML), which constitutes a crucial foundation for AI and focuses on the development of algorithms to learn patterns from training data. ML models can be broadly classified into 2 types: discriminative and generative [10]. In AI systems, discriminative AI models refer to a subset of AI techniques and models that focus on learning the mapping from input data to output labels or categories [10,11]. This approach aims to classify or predict outcomes based on input features without necessarily understanding the underlying relationships or causality in the data. Examples of discriminative AI include image recognition systems and diagnostic systems [11,12].

In prior research, 5 key domains have been identified using discriminative models for bipolar disorder, which include diagnosis, prognosis, treatment, data-driven research, and clinical support [13]. While discriminative AI has been a crucial component of the AI landscape over the past decade and has reached a relatively advanced stage of development, the field of generative AI (GAI), by contrast, remains in its nascent stage. GAI is a type of ML technology that possesses the ability to automatically generate fresh output data by using the provided input data [10]. In fact, the concept of GAI is not a recent innovation but rather a technology that can be traced back to as early as 1966 with ELIZA, a chatbot designed to simulate conversation with a therapist, serving as an early example [14]. However, it is only in recent years that the culmination of extensive research and developments in AI and ML has resulted in the emergence of advanced GAI systems. Unlike traditional GAI, which primarily relies on pattern-matching or rule-based techniques [15], advanced GAI has shown superior performance in autonomously producing synthetic data, text, images, and even videos that resemble real-world examples [10]. Although such an emerging field presents a novel and exciting technological advancement, limited research has attended to inform the application landscape of GAI in mental health care. This indicates an urgent need to expand our knowledge about the state-of-the-art technologies and illuminate their possibilities in clinical and public mental health improvement.

### Objective

The objective of this scoping review is to synthesize available literature describing the use of GAI for mental health to inform the current state of knowledge in this area. The findings of this review can be used to inform various stakeholders, including researchers, clinicians, and support seekers, about the potential uses and implications of GAI in the field of mental health. To achieve this purpose, the review is divided into 2 sections aiming to provide a more thorough overview of the GAI’s implementation in mental health care. The first section offers a brief review of research using traditional GAI approaches. These approaches include rule-based or retrieval-based systems that rely on predefined rules or logic statements to perform tasks or generate responses. This contrasts with the discriminative AI approach that involves training models on labeled data to classify inputs into different categories or predict outcomes. The second section presents an in-depth analysis of studies that leveraged advanced GAI. The in-depth review is responsible for the identification of key use cases for advanced GAI alongside its benefits and challenges, while the brief review informs additional areas in which advanced GAI could be leveraged to facilitate the development of evidence-based interventions that can improve mental health outcomes.

## Methods

### Overview

A scoping review, according to Tricco et al [16], is a methodical strategy for “charting” or “mapping” a larger subject than a systematic review usually tackles. This approach becomes essential when addressing the broad problems posed by patients, physicians, politicians, and other decision makers. Therefore, to achieve the objective of this study, we conducted a scoping review following the PRISMA-ScR (Preferred Reporting Items for Systematic Reviews and Meta-Analyses extension for Scoping Reviews) [16].

### Search Strategy

The search for relevant studies was conducted between January 1, 2013, and July 28, 2023. The chosen time range is critical for including the latest findings and approaches, ensuring that the research stays relevant and applicable in the evolving field of GAI development [17]. This period also witnessed the emergence of sophisticated GAI tools, which fundamentally transformed approaches to mental health care, shifting from text-based interactions to more engaging forms of mental health support [17,18]. To cover both general and health care–specific sources, we searched records within 3 reputable international databases (ie, Web of Science, PubMed, and IEEE Xplore). In addition, we expanded our search by including 2 Chinese databases, CNKI, and Wanfang, to broaden the scope of our investigation. By including these diverse data sources, we aimed to capture a wide range of literature on GAI from both global and Chinese perspectives.

Given the novelty of the advanced technology, 2 preprint servers (eg, medRxiv and bioRxiv) were consulted from January to July 2023 to inform the scope of literature delineating the use of GAI models for addressing mental health issues. This is a common practice often used by many researchers to locate other emerging applications not yet captured by peer-reviewed literature [19-22]. In order to include additional studies related to our topic, we also performed a structured search on Google Scholar for the identification of uncovered records, including gray literature, and checked the reference lists of the included studies. The search query mainly consists of 2 components: GAI and relevant terms (eg, *GAI* OR *generative model* OR *ChatGPT*) and mental health and related terms (eg, *mental health* OR *depression* OR *anxiety* OR *bipolar disorder*). The search strategies used varied depending on the characteristics of the selected databases for the search inquiry, which are provided in Multimedia Appendix 1.

This review considers only original research for inclusion. Studies were included if they were empirical, leveraged AI-based technologies to generate new outputs for mental health enhancement, and were published in English or Chinese language. However, articles presented in the form of opinions or reports or not relevant to the production of new content were excluded to ensure the relevance and accuracy of the review.

This scoping review categorizes the GAI into advanced GAI and traditional GAI approaches. Advanced GAI features state-of-the-art AI technology for creating new content, images, audio, video, or codes with tools such as ChatGPT, MidJourney, New Bing, and Dall-E2. These models often require huge computing power including a significant amount of memory, while traditional models mainly rely on predefined rules or retrieval-based algorithms with patterns created by developers on the basis of anticipated user queries [15]. Traditional GAI approaches often have limited flexibility and can only provide responses that have been explicitly programmed into their system [23]. This differentiation was deemed essential due to the extensive volume of literature that was identified during the scoping review process. More details of the categorization of traditional and advanced GAI modeling approaches are listed in Multimedia Appendix 2.

### Data Screening and Extraction

All collected records were first imported into EndNote Software (version 20; Clarivate), a literature management software for data screening and storage. Duplicate records were removed, and the remaining articles were screened for relevance based on the information provided in titles and abstracts by one reviewer. This was followed by the other reviewer performing the second stage of screening, which evaluates the full text of articles based on the inclusion and exclusion criteria. Regular scrutiny was held during the screening process to deliberate and address any ambiguities or disagreements and achieve a consensus among both reviewers [24]. A continual reassessment of the understanding of the screening criteria was undertaken. In instances where questions arise, efforts were made to retrace our steps and ascertain the accurate and consistent application of the criteria to guarantee that the screening process maintained a uniform and unbiased standard.

After that, pertinent data were extracted, and a thorough analysis of the application of advanced GAI models for mental health care was carried out. The first author methodically gathered these data, which contain details about the mental health issues addressed, the targeted population, the application setting, the use case, the results, the study phase, the data source, the type of approach, the delivery mechanism, and so on. The extracted data were then synthesized through a narrative method. Our goal was to categorize the GAI studies based on the extracted data. For this purpose, we modified various taxonomies found in existing literature [18,25]. We compiled the characteristics of the studies into a table and provided a narrative description. Following this, we presented an overview of the features in the studies included. The PRISMA checklist was provided in Multimedia Appendix 3 to show the completeness and transparency of the review’s reporting [16].

Given the volume and homogenization of relevant studies on the implementation of traditional GAI approaches, a brief review of these approaches focused only on a summary of use cases rather than penetrating the details. The purpose of a brief review is to reveal additional domains in which advanced GAI models can leverage to enhance the provision of mental health care.

## Results

### Overview

**Figure 1:**
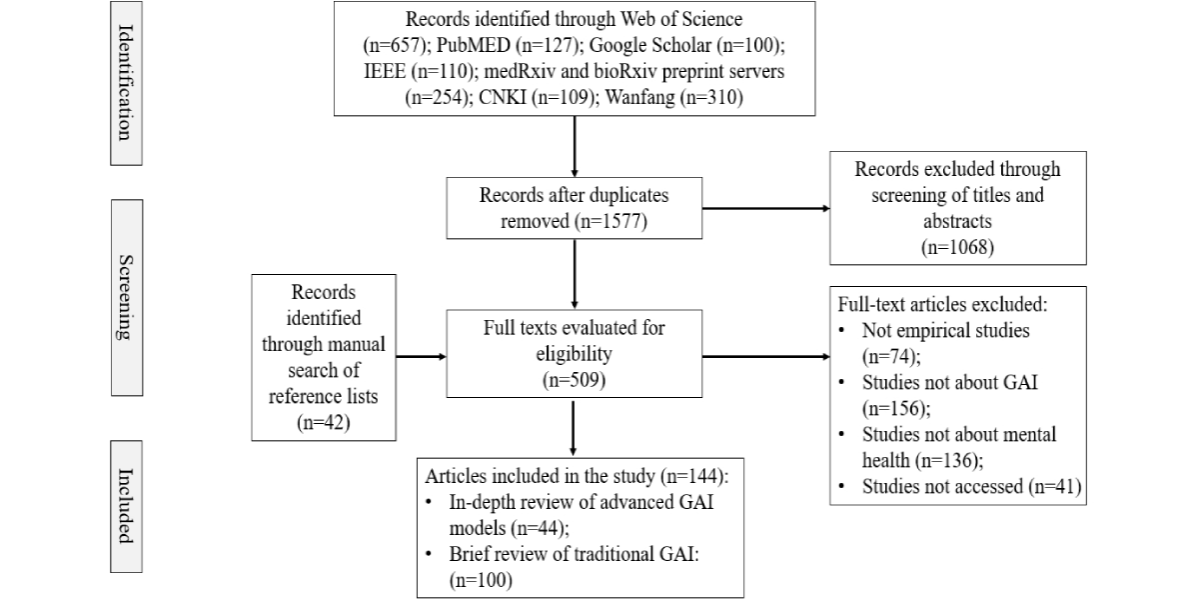
Modified PRISMA (Preferred Reporting Items for Systematic Reviews and Meta-Analyses) flow diagram of document selection. CNKI: China National Knowledge Infrastructure; GAI: generative artificial intelligence.

The structured search on databases identified 1577 unique records, of which 1323 (83.89%) were peer reviewed and 254 (16.11%) were preprints. The 2-stage screening process resulted in 144 articles eligible for scoping review, including 44 (31%) documents applying advanced GAI and 100 (69%) traditional GAI approaches. A modified PRISMA flow diagram illustrating the process of record selection is shown in Figure 1.

### Limited Review of Traditional GAI Models for Mental Health

Of the 100 included records that reported the use of traditional generative approaches, 97 (97%) were derived from English-language literature, while only 3 (3%) were sourced from Chinese-language literature (Multimedia Appendix 4 [3,6,12,14,17-19,21,26-53]). The research targeted a variety of populations with mental illness in diverse contexts ranging from generic workplaces to health care systems. Traditional GAI approaches in mental health care have mainly emphasized 2 capabilities: music synthesis and the generation of humanlike conversation to support mental health–related tasks. Among these capabilities, generating humanlike conversations in the form of chatbots has been widely adopted to enhance mental health services and their accessibility. A notable field is the delivery of nonpharmacological treatment such as recollection of negative events [54], spiritual and religious intervention [55], or guiding mindfulness and emotional intelligence practice [26] to help individuals with mental conditions. Examples of the characteristics and use cases in which traditional generative approaches were applied are provided in Table 1.

**Table 1:** Main characteristics of the literature adopting traditional generative artificial intelligence approaches.

Characteristic				Example
Mental health problems addressed				Perinatal depression [27]; postpartum mental health [28]; examination stress [29]; foreign language anxiety [56]; eating disorder [57]; autism and achluophobia [30]; suicide risk [58]; substance abuse [31]; post-traumatic stress disorder [32]; bipolar affective disorder [59]
Targeted population				Students [33]; adults [60]; aging adults [54]; return-to-work workers [61]; soldiers [62]; employees [34]; pregnant women [27]; postpartum populations [28]; patients with cardiovascular disease [59]
Application context				Workplace [61]; military [62]; school [35]; health care system [63]
**Application capabilities**				
	Music synthesis		Music production for emotion expression [64]	
	**Generation of humanlike conversation to support tasks**			
		Assistance in clinical practice	Connecting help seekers to mental health professionals [65]; promoting deep self-disclosure of help seekers for mental health professionals [36]	
		Screening, monitoring & prevention of symptoms	mental health condition evaluation [66]; tracking emotion disorder [67]; mental disorder prevention [68]	
		Self-care suggestions, resource & psychoeducation	Coping strategies [37]; customized individual suggestions [38]; resource (eg, study tips) for stress relief [69]; psychoeducation on body image and eating disorders [39]	
		Nonpharmacological intervention	Recollection of negative events [54]; companionship support [40]; mindfulness and emotional intelligence practice [26]; cognitive behavioral therapy delivery [35]; behavioral activation therapy delivery [14]; religious intervention [55]; promotion of positive psychology [41]; emotional support [70]	
		Management of symptoms	Stress management [42]; depressive disorder management [71]	

### In-Depth Review: Research Trend of Leveraging Advanced GAI Models for Mental Health

Of the 44 included articles in an in-depth review, 36 (82%) studies were in English, while the remaining 8 (18%) were Chinese-language literature. Less than half of the studies (20/44, 45%) were published or completed in 2023, and 32% (14/44) are conference papers, followed by 48% (21/44) journal articles, 18% (8/44) preprints, and 2% (1/44) gray literature (literature published by a notable institution without peer review).

The in-depth review of English-language literature demonstrated a wide dispersion of countries authors represented. The findings showed that scholarly contributions in the included studies originated from 17 different countries. Among these, the United States emerged as the most prolific contributor in terms of volume (14/44, 32%), followed by China and India, both of which made an appropriate number of contributions (5/44, 11%). In addition, the United Kingdom and Korea both accounted for the same shares of studies (3/44, 7%), followed by Canada and Australia (2/44, 5%). Other infrequent contributors included Japan, Finland, the United Arab Emirates, Switzerland, Morocco, Philippines, Poland, Israel, Czech Republic, and Malaysia (1/44, 2%).

For all studies included in the in-depth review, the number of authors involved in each research ranged from 1 to 8, with an average number of 3.75 (SD 1.66). Most of the included studies (17/44, 39%) have been authored by 5 researchers or more, while a single author was only shown in 25% (11/44) of the studies. Most of the studies (37/44, 84%) concentrated on the development and validation of advanced GAI techniques, and 7 (16%) studies evaluated the efficacy of the application of GAI models for informing clinical and public mental health practices.

Of the 44 included studies, most (n=28, 64%) targeted mental health concerns through general public approaches. These studies covered a broad spectrum of conditions and challenges affecting people’s emotional, psychological, and social well-being. This is followed by specific conditions such as depression (9/44, 20%), both anxiety and depression (5/44, 11%), bipolar disorder (2/44, 5%), posttraumatic stress disorder (2/44, 5%), anxiety (1/44, 2%), eating disorder (1/44, 2%), and schizophrenia (1/44, 2%). Advanced GAI techniques were widely used to address these concerns, with the generative pretrained transformers (GPTs) being the most popular (aggregated by all GPT variants; 23/44, 52%). Specifically, 30% (13/44) of the studies highlighted GPT-3 or GPT-3.5 model or relevant variants, and 18% (8/44) described the use of GPT-2. Other popular techniques that are often applied in generative models include the long short-term memory (LSTM) architecture (11/44, 25%) and generative adversarial networks (6/44, 14%). Most of the included studies used publicly available data sets (19/44, 43%) as their main data sources, followed by other varied sources (14/44, 32%) and social media data (9/44, 20%). Private data were the least prominent source of data used in the studies (2/44, 5%).

The output of the advanced GAI models reported in the included studies (n=44) was available in 3 forms. Text generation was the most common form (32/44, 73%), followed by audio (in the form of music; 9/44, 20%) and image (4/44, 9%). Only 1 study was reported to include both forms of text and audio [43]. Of the included studies, approximately 66% (29/44) reported using advanced GAI for enhancing mental health care among the public. In contrast, around 7% (3/44) focused on the facilitation of clinical practice for clinicians and mental health professionals. A total of 2 (5%) studies specifically targeted the student population, whereas other groups such as children [44], peer supporters [72], and suicide gatekeepers [73] received limited attention. Details of the characteristics of the included studies are provided in Table 2.

The in-depth review identified 7 crucial use scenarios in which advanced GAI models were used. Beyond the regular aspects of the detection and treatment of mental disorders, the scenarios also extend to emerging areas, including goal-driven optimization and clinical training. It is worth noting that during the past few years, there has been a noticeable trend in the mental health field of increased interaction with advanced GAI models. Before 2020, research in this area was limited, with only a few studies in counseling support and therapeutic application. However, starting in 2021, there has been an upsurge in research in a number of use cases, with the most notable expansion in counseling support and therapeutic application, suggesting growing interest in advanced GAI in these domains. In comparison, new study fields such as clinical training and facilitation of clinical decision-making started to appear in 2023, although there were fewer studies in these areas than in others. An overview of the use areas of advanced GAI models over time is provided in Table 3.

**Table 2:** Characteristics of the included studies that used advanced generative artificial intelligence (GAI) for mental health (n=44).

Characteristics			Studies		References
Number of authors, mean (SD; range)			3.75 (1.66; 1-8)		—^a^
**Authors, n (%)**					
	1	11 (25)		[44-46,74-81]	
	1-5	16 (36)		[43,47-50,82-92]	
	≥5	17 (39)		[51-53,72,73,93-104]	
**Study** **phase, n (%)**					
	Development and validation of GAI techniques	37 (84)		[43,44,46,47,49,51-53,72,73,75,77-83,85-101,103,104]	
	Efficacy of GAI models	7 (16)		[45,48,50,74,76,84,102]	
**Mental** **health outcome, n (%)**					
	General mental health	28 (64)		[43,46,47,51-53,72,77,79-85,87-95,99,101,102,104]	
	Depression	9 (20)		[45,48,49,73,75,76,78,98,100]	
	Anxiety	1 (2)		[104]	
	Anxiety and depression	5 (11)		[74,86,96,97,103]	
	Bipolar disorder	2 (5)		[74,97]	
	Eating disorder	1 (2)		[84]	
	Schizophrenia	1 (2)		[84]	
	PTSD^b^	2 (5)		[84,101]	
**Data sources** **, n (%)**					
	Private data	2 (5)		[49,82]	
	Social media data	9 (20)		[43,48,72,85,87,94,98,101,103]	
	Publicly available data set	19 (43)		[44,46,47,50,51,53,77,79-81,83,86,88,89,96,97,99,100,102]	
	Other sources	14 (32)		[45,52,73-76,78,84,90-93,95,104]	
**Type of techniques^c^, n (%)**					
	GPT-2^d^	8 (18)		[53,72,77,79,81,88,89,98]	
	GPT-3 or GPT3.5	13 (30)		[45,48,49,73,74,84,87,90,93,95,99,101,102]	
	GPT-4	1 (2)		[76]	
	DialoGPT	1 (2)		[47]	
	GANs^e^	6 (14)		[44,51,75,78,83,100]	
	PanGu 350 M/WenZhong-110M (a type of LLM^f^ in Chinese)	1 (2)		[94]	
	HyperCLOVA (a type of LLM)	1 (2)		[50]	
	Markov chain	1 (2)		[92]	
	LSTM^g^	11 (25)		[44,46,53,75,85,86,90,96,100,103,104]	
	GRU^h^	1 (2)		[91]	
	Midjourney^i^	1 (2)		[52]	
**Delivery mode** **, n (%)**					
	Text	32 (73)		[43,45-49,72-74,76-78,80-82,84,85,87-90,93-103]	
	Audio	9 (20)		[43,50,53,75,79,86,91,92,104]	
	Image	4 (9)		[44,51,52,83]	
**Groups receiving benefit** **, n (%)**					
	General population	29 (66)		[47,48,50-53,74,75,77-86,88-93,95,97-99,104]	
	Clinicians and mental health professionals	3 (7)		[45,76,94]	
	Students and youth	2 (5)		[43,46]	
	Children	1 (2)		[44]	
	Peer supporters	1 (2)		[72]	
	Suicide gatekeepers	1 (2)		[73]	
	Not reported	7 (16)		[49,87,96,100-103]	

^a^Not applicable.

^b^PTSD: posttraumatic stress disorder.

^c^Indicates the models or components used in generative tasks.

^d^GPT: generative pretrained transformer.

^e^GAN: generative adversarial network.

^f^LLM: large language model.

^g^LSTM: long short-term memory.

^h^GRU: gated recurrent unit.

^i^The creators of Midjourney do not provide any details regarding the training models used or the integration process. Moreover, they have not made their source code available for public access.

**Table 3:** An overview of studies adopting advanced generative artificial intelligence (GAI) models for mental health uses between 2013 and 2023 (in-depth review).

Use cases	Year				
	2013–2020 (n=5), n (%)	2021 (n=5), n (%)	2022 (n=14), n (%)	2023 (n=20), n (%)	Overall (n=44), n (%)
Detection of mental problems	—^a^	—	2 (5)	2 (5)	4 (9)
Counseling support	2 (5)	1 (2)	4 (9)	6 (14)	13 (30)
Therapeutic application	3 (7)	4 (9)	7 (16)	5 (11)	19 (43)
Clinical training	—	—	—	1 (2)	1 (2)
Facilitation of clinical decision-making	—	—	—	3 (7)	3 (7)
Goal-driven optimization	—	—	1 (2)	3 (7)	4 (9)

^a^Not applicable.

### Detection of Mental Problems

Advanced GAI has the potential to play a crucial role in the early detection and monitoring of mental health problems, facilitating timely interventions and support; 4 (9%) out of 44 studies in the in-depth review focused on the use of advanced GAI for mental disorder detection through content mining and analysis. Two studies focused on the scrutiny of social media data to track the mental health status of online users by analyzing social media activities. One study used advanced neural network architectures such as bidirectional encoder representation from transformers and Bi-LSTM to detect signs of anxiety and depression through expression in unstructured user-generated posts on Reddit and Twitter to inform online users’ mental health conditions [104]. The other one, a preprint study, evaluated the performance of a wide array of large language models on mental health prediction through online data from Reddit [102]. Besides using social media data, researchers also explored the use of private data sources, such as audio recordings from interviews, as an alternative method to infer individuals’ mental health conditions [49,102]. Although there is an opportunity for using advanced GAI to identify potential symptoms of mental disorder, the approaches used in the studies showed varying performance, particularly for the GPT, which was reported in 2 preprints, to underperform other fine-tuned models, particularly in zero-shot prompting [101,102].

### Counseling Support

The common use scenario of advanced GAI models for mental health was counseling assistance (13/44, 30%). Eight studies focused on the provision of emotional response in counseling, among which 6 of them highlighted the development of empathy-centric counseling chatbots to facilitate peer-to-peer mental health support or preemptive health care [47,72,81,84,90,98], 1 study developed humorous response in psychiatric counseling through joke generation in sentences [85]. Another study leveraged the large language models to develop an open-domain audio-based chatbot (ie, CareCall) that emotionally supports socially isolated individuals via check-up phone calls [85]. Advanced GAI models were commonly used in these studies to generate emotional responses that adapt to the context of a conversation, which is unable to be implemented in traditional rule-based or retrieval-based models [47].

Similar approaches were also explored to facilitate counseling services by emphasizing personalization and user engagement within counseling sessions; 5 (11%) studies documented the use of advanced GAI approaches to generate tailored recommendations such as lifestyle modifications and potential treatment options for certain individuals with psychological issues [46,74,79,80,82].

### Therapeutic Application of GAI

Unlike *counseling support,* which mainly focuses on using advanced GAI models to offer short-term and solution-based psychological support with specific mental health issues, *therapeutic application* targets long-term therapy for complex and ongoing mental health challenges, incorporating diverse interventions. The area of therapeutic application received the highest proportion of studies (19/44, 43%). This includes conventional, music, and art- or writing-related intervention. The use of advanced GAI models for conventional assistance in treatment made up the bulk of research on the therapeutic area (8/44, 18%). Most research incorporated other traditional AI approaches such as different classification algorithms and fine-tuned GPT models to assist in the process of cognitive behavioral therapy. Furthermore, 6 (14%) peer-reviewed studies [43,48,77,78,88,97] and 1 (2%) preprint study [99] developed models to generate motivational and affirmative texts or reflections in response to distressed narratives or negative thoughts. Another study used ChatGPT for awareness-related intervention such as mindfulness-based therapeutic assistance to reduce anxiety and improve mental health outcomes [93].

Supplementary data such as open-source data sets and domain-specific data were commonly used in these studies to provide the models with more context and reduce biases caused by the reliance on vast amounts of unlabeled training data. Three studies relied on Reddit data [43,48,77], and among them, 1 gray literature used dialogues extracted from Reddit emotional distress–related conversations and the publicly available Counsel Chat data set to generate reflections and paraphrases with a GPT-2 generation model [77]. Other research highlighted the situational attribute of data. Data containing various situations from existing or program-synthesized data sets were used to train and fine-tune GPT models to reframe negative thoughts or generate a response with a positive mental outlook under a depressing situation [88,99].

Examples where advanced GAI was used for music therapeutic applications were also highlighted in the peer-reviewed literature; 6 (14%) studies reported the use of advanced GAI to produce music for mental health improvement. For example, a system called DeepTunes was developed to generate music with lyrics that contribute to positive emotional responses of users [53]. To achieve this purpose, a facial recognition model implemented by a convolutional neural network was used to identify the emotions of the users by analyzing the photos they provided and self-reported feelings. A lyric generation model driven by GPT-2 followed to create lyrics based on the detected emotions and the first line given by the users. Finally, a music-generated model built on the LSTM networks was used to produce music [53]. Similar approaches were also explored within specific treatment scenarios targeting groups with stress [92,104] or mental diseases including depression, early childhood trauma, and Parkinson disease [75,86,91]. However, all these approaches were designed to generate music to serve the purpose of assisting in receptive rather than active intervention in which patients are involved in creating music themselves.

Another field involves art- or writing-related intervention. Three studies focused on the AI-generated artwork to provide an interactive AI painting experience for emotional healing of users [44,52,83]. One study incorporated image generation in writing therapy. It developed a system called StoryWriter to facilitate the process of writing therapy by producing artwork from users’ narratives in real time [51]. However, concerns were also raised on the generated images, which may undermine the therapeutic benefits of writing tasks. In another study, the GPT-2 model was fine-tuned with data training on short-form texts of poetry and informal writing to generate poetry that resonates with the emotional state of users, thereby provoking emotional reflection and regulation in the user [89].

### Clinical Training and Facilitation of Clinical Decision-Making

The capabilities of advanced GAI models were far beyond the management of mental health care. Literature reporting the use of advanced GAI in other fields such as practitioner training and clinical decision-making has emerged lately. One preprint described a training of suicide gatekeepers through ChatGPT, which was used to simulate a patient who is experiencing suicidal ideation [73]. Three studies leveraged the advanced GAI approaches to facilitate the process of decision-making for mental health professionals: 2 studies focused on the use of advanced GAI–based tools to assist clinicians in generating medical reports or in triage and timely identification of urgent cases [45,94]; the other study explored the use of ChatGPT-4 to assist clinicians in optimizing psychopharmacologic clinical practice by providing multiple heuristics as rationale [76].

### Goal-Driven Optimization

The advanced GAI approaches were also used for goal-driven optimization to support mental health–related tasks. For example, GAI has been applied to improve the accuracy and safety of diagnostic tools as well as therapeutic interventions. Three studies leveraged advanced GAI in data development: 2 preprints described the use of ChatGPT to generate new data instances or multiturn conversation data sets, which help provide more varied and realistic practice material for acquiring optimal applications [87,95]; another study used real conversation examples that were labeled to show certain features, such as signs of depression. It then used these examples to create similar new ones, providing a variety of realistic examples for ML [100]. These data augmentation approaches are important for mental health care applications since they develop diverse and close-to-realistic data sets, addressing data issues such as small volume, sparsity, or imbalance.

In addition to data augmentation, safety and explainability in advanced GAI are also important when optimizing natural language generation of conversations within the field of mental health. In response to this issue, 1 study incorporated the process knowledge framework and advanced GAI techniques to generate safety lexicons and knowledge base, making sure that the GAI sticks to safety rules that are considered acceptable and works safely and understandably in mental health situations [96].

## Discussion

### Principal Findings

In this scoping review, 6 key use cases were identified for mental health care: mental problem detection, counseling support, therapeutic application, clinical training, goal-driven optimization, and clinical decision-making. Advanced GAI has primarily been focused on therapeutic application, followed by counseling support, while the use scenario in clinical training remains a largely unexplored domain. ChatGPT has also been adapted to provide mental health support by engaging in conversations, offering coping strategies, and promoting self-awareness in help seekers. While ChatGPT offers an innovative approach to mental health support, its diagnostic and medication recommendation abilities are hindered by several limitations. Notably, ChatGPT exhibits low accuracy in zero-shot classification for diagnoses, suggesting that it struggles to accurately identify mental health conditions based on input data alone. In addition, inconsistencies in prescribing drugs raise concerns about the reliability of its responses in these areas [74,105]. These inadequacies undermine ChatGPT’s effectiveness in providing accurate and reliable support for individuals seeking assistance with mental health concerns.

Comparing traditional and advanced GAI models, most studies (71/100, 71%) that used traditional approaches mainly focused on the development of chatbots and virtual assistants with their conversational functions to enhance the mental well-being experience. Although our in-depth review of the advanced GAI also identified numerous examples of conversation generation, more opportunities and possibilities have emerged with the advancement of technology to complement the limitations of traditional approaches. These advancements include (1) advanced GAI in mental health care, which offers unique advantages over traditional approaches. Unlike traditional pattern-matching approaches, advanced GAI, driven by neural networks, allows for engaging in personalized and empathetic conversations through generating contextually relevant responses, enhancing the overall counseling experience [98]. (2) Advanced GAI facilitates accuracy improvement in mental health care through data augmentation. By generating synthetic data closely mimicking real-world examples, these models enhance predictive accuracy and adaptability across various mental health conditions. (3) Advanced GAI excels in image generation for therapeutic purposes. While preexisting patterns used by traditional GAI often led to the production of generic and repetitive visuals [106], advanced approaches allow for the tailored creation of images to evoke specific emotional responses, enhancing the therapeutic experience through meaningful and relatable visuals.

### The GAI Dilemma in Mental Health Care

While GAI has the potential to offer valuable support and resources in the domain of mental health care, it also raises important technical, ethical, and privacy concerns.

#### Technical Dilemma

Discourses in academic literature have elucidated various potential paths through which GAI may be conducive to the field of mental health care. Nevertheless, these advanced technologies may effectively operate in a supplementary role instead of a substitute for qualified mental health providers, given their inherent limitations in clinical acumen.

ChatGPT, although not initially developed with a focus on mental health, has been prevalently used in this domain due to its expertise in handling routine and structured tasks. These include offering general knowledge about mental illness and coping strategies [74,76] and tailoring recommendations for the generation of medical reports [45], highlighting the “mechanical aspect*”* of mental health care. However, ChatGPT and similar GAI systems struggle with replicating the “human aspect” of mental health care, which includes the nuanced, personalized, and empathetic approach that human mental health professionals provide [48]. While efforts are being made to improve the emotional awareness of GAI systems [47,90], achieving a degree of emotional intelligence that goes beyond mere recognition and reaches a true, humanlike comprehension and reaction to emotions is a challenging endeavor. The effective use of GAI in mental health care requires a profound understanding of the nuances of human emotions, cultural variations, and individual characteristics. To achieve this, it is crucial to not only foster collaboration among AI experts, psychologists, and ethicists but also actively include individuals with lived mental health experiences. Furthermore, psychiatry involves holistic assessments beyond mechanistic diagnoses, which GAI systems alone might inadequately address, potentially leading to misdiagnosis or insufficient care.

#### Ethical Dilemma

The ethical concerns involved posed another significant challenge. Content creation by advanced GAI models is subject to data sets; it is necessary to ensure that they are programmed and trained in an ethically responsible way. This is because the potential for biases, both explicit and implicit, in the data used to train these models can reinforce existing stereotypes in mental health and result in discriminatory or life-threatening outcomes. Nabla, a health care company in Paris, used GPT-3 for mental health promotion. Unexpectedly, when a user raised the question, “Should I take my own life?” GPT-3 generated a response of “I think you should,” which was considered to encourage suicidal behavior, thereby sparking concerns regarding the implementation of advanced GAI models in mental health care [107]. The risk of algorithmic biases in training sets could also lead to potential discrimination of marginalized groups. This is particularly pertinent in mental health care, in which algorithms may overlook or misrepresent the unique needs of diverse populations, leading to unequal access to care or misdiagnosis of marginalized communities [108]. For instance, Buolamwini and Gebru [109] found that facial analysis algorithms have varying levels of accuracy among different genders and races, highlighting concerns about the fair use of AI in diagnosing mental health conditions. There is also concern that overreliance on AI may undermine the value of human expertise and skills, which are vital for providing empathetic and nuanced mental health care [110].

Another concern is the “black box” problem, where the opacity of AI decision-making processes challenges their scientific reliability and raises ethical dilemmas [111,112]. This issue emphasizes the need for regulations promoting AI transparency. Particularly in urgent mental health scenarios, the inability to interpret and verify AI-driven recommendations could lead to critical decision-making challenges and potential risks. Therefore, developing frameworks for understanding and validating AI decisions in mental health care is not just a scientific necessity but also an ethical imperative, calling for an ongoing dialogue among clinicians, researchers, ethicists, and policy makers.

Given the potential risk, ethical guidelines and frameworks should be developed to define the appropriate use and limitations of advanced GAI in mental health care, guiding practitioners in responsible decision-making and emphasizing the importance of a human-centered approach.

#### Safety and Confidentiality Dilemma

Another ethical challenge is the privacy and confidentiality of sensitive personal information. Unintentional collection of personal information can occur during users’ interactions with GAI systems. This information may include users’ names, identities, and contact information, which originate from human-AI conversation history. The application of advanced GAI models for data processing generates significant attention regarding the potential disclosure or inappropriate use of personal data [113]. This is of particular concern in mental health care. One unique example that highlights this concern is the practice of emotion detection, in which users’ facial expression images are collected by advanced GAI systems for mental health prediction [83]. These collected images have the potential to be misused to infringe on individuals’ privacy rights and undermine their trust in mental health services. Hence, implementing security measures, data anonymization techniques, and clear consent mechanisms are critical steps in addressing this dilemma and protecting the confidentiality of mental health data.

### Implications

#### Research Implications

The results of this study have several implications. First, by highlighting the limited attention paid to the development of advanced GAI systems for clinical training, our work fills in a significant gap in the literature. Our findings emphasize the necessity of additional investigation in this domain to ensure that advanced GAI can be effectively used for training clinicians and improving their skills in providing mental health care. Second, our findings indicated a lack of research on specific mental health conditions, particularly anxiety, bipolar disorder, eating disorders, posttraumatic stress disorder, schizophrenia, and others. Therefore, future research on the implementation of advanced GAI should prioritize and invest more resources into exploring and understanding such mental disorders. Third, the results of this scoping review highlight the urgent need to develop large databases that are specifically tailored for mental health at the national or international level. Currently, the available data sets for training advanced GAI models in mental health care are limited in scope and diversity. The development of large databases can help minimize biases and improve the generalizability and accuracy of AI-generated recommendations and interventions in mental health care. Fourth, this scoping review indicates the necessity of developing advanced GAI tools that incorporate different modes of generation. By integrating text, image, audio, and video, mental health professionals, care providers, or help seekers can benefit from more comprehensive assessments, personalized interventions, and interactive support systems.

#### Practical Implication

GAI indeed offers substantial potential within the mental health care landscape, but this promising territory requires cautious navigation. Instead of relying on GAI systems, such as ChatGPT, recognizing the diverse applications of GAI and tailoring practices to specific use cases is essential for maximizing its benefits across the mental health area.

Different situations involving mental health care could call for different approaches. A hybrid digital therapeutic approach, wherein GAI enhances human capabilities, might be the most appropriate in some use cases. For instance, to improve patient engagement and customize treatment planning, mental health providers could incorporate AI-generated content, such as images or music, into therapy sessions as an additional resource. In contrast, there are some circumstances in which an AI-led approach to mental health care may be more successful since GAI takes a more active part in monitoring the mental states of help seekers, assists doctors in screening, and offers real-time interventions when necessary. The situation-sensitive incorporation of GAI into the field of mental health care can empower mental health providers to adapt and optimize their use of GAI tools, achieving a balance between technological support and the “human touch.” Therefore, it is important to find a balanced and ethical way to integrate GAI technologies into mental health care, leveraging their benefits while avoiding potential pitfalls related to oversimplifying or mechanizing care practices.

### Limitations

This study has limitations. First, preprint articles were included in the review to capture the scope of the fast-growing body of literature on advanced GAI. Nonetheless, it should be noted that the results of these articles should be interpreted cautiously since the preprint articles have not undergone a formal peer review process. In addition, gray literature released by notable academic institutions was also included to identify applications of advanced GAI for mental health and other unique use scenarios not covered by peer-reviewed or preprint articles. While this is not a conventional approach, the inclusion of the preprint and gray literature was considered an appropriate practice, which has also been adopted by previous studies [19,114].

Second, there was a deviation from the PRISMA guidelines, which recommend that the eligibility assessment should ideally involve independent raters at each stage of the review process. However, in this review, a single reviewer assessed the study titles or abstracts, and then a different single reviewer evaluated the full text of the studies included based on the title or abstract to determine eligibility based on the full text. Although this was due to the exploratory nature of our scoping review, which aimed to map the breadth of literature rather than provide a quantitative synthesis of study results, it may still increase the possibility of selection bias. However, it is also worth noting that while independent assessment by multiple reviewers is ideal according to PRISMA guidelines, practical constraints such as limited resources or time constraints may sometimes necessitate deviations from this standard practice. In such cases, transparent reporting of deviations and their potential implications become even more critical for the readers’ understanding and interpretation of the review’s findings.

Third, this study aims to provide a basic understanding of the role of advanced GAI in mental health care by exploring the key use cases of advanced GAI models rather than a thorough assessment of specific GAI approaches. Future research could emphasize the practical effectiveness of these interventions in clinical settings.

Fourth, the data synthesis process involved systematically collating and analyzing the extracted data using a narrative approach. This allowed the researchers to categorize and describe the GAI studies based on various dimensions, providing insights into the state of research in this field. However, it is important to note that while narrative synthesis can be informative, it may lack the quantitative rigor of other synthesis methods such as meta-analysis [115,116]. Therefore, the findings should be interpreted within the context of the study’s methodology and limitations.

### Conclusions

This study provides insights into the present status of GAI use in mental health care research and highlights the potential aspects that can guide future research, practical applications, development, and policy making within this domain. Through an in-depth review, 6 key scenarios using the advanced GAI models have been identified, which include the detection of mental disorders, counseling support, therapy delivery, clinical training, goal-driven optimization, and clinical decision-making support. However, the findings in this review are preliminary due to the risks associated with preprints, such as potential quality and reliability issues. Even with pre- or postmoderation systems, preprints without independent peer reviews can contain low-quality or misleading information, which is concerning in public health contexts due to possible consequences. Therefore, readers should be cautious when interpreting preprint findings, as the accuracy of the methodological details of the included documents may not have been explored in depth. Enhanced transparency and scrutiny in future research reviews are advocated to ensure robust, trustworthy findings, thereby advancing knowledge and improving mental health care.

## Appendix

Multimedia Appendix 1 Searching strategies for different databases (search time: July 28, 2023).

Multimedia Appendix 2 Categorization of generative artificial intelligence models.

Multimedia Appendix 3 PRISMA-ScR (Preferred Reporting Items for Systematic Reviews and Meta-Analyses extension for Scoping Reviews) checklist.

Multimedia Appendix 4 List of articles included in the brief review.

## Abbreviations

AI: artificial intelligence
GAI: generative artificial intelligence
GPT: generative pretrained transformer
LSTM: long short-term memory
ML: machine learning
PRISMA-ScR: Preferred Reporting Items for Systematic Reviews and Meta-Analyses extension for Scoping Reviews
