# Supplementary material for: Debate and Dilemmas Regarding Generative AI in Mental Health Care: Scoping Review
Source: Interact J Med Res. 2024 Aug 12;13:e53672. doi: 10.2196/53672 (PMC11347908; doi:10.2196/53672)
Supplement: Multimedia Appendix 1 [file ijmr_v13i1e53672_app1.docx]

Multimedia Appendix 1. Searching strategies for different databases (search time: July 28, 2023)

| PubMED | ((("Artificial Intelligence"[Mesh]) OR ((((((((((((("Artificial Intelligence"[Title/Abstract]) OR ("AI"[Title/Abstract])) OR ("neural network"[Title/Abstract])) OR ("machine learning"[Title/Abstract])) OR ("supervised learning"[Title/Abstract])) OR ("unsupervised learning"[Title/Abstract])) OR ("deep learning"[Title/Abstract])) OR ("generative AI"[Title/Abstract])) OR (LLMs[Title/Abstract])) OR ("large language model*"[Title/Abstract])) OR ("AI generat*"[Title/Abstract])) OR (GANs[Title/Abstract])) OR (VAEs[Title/Abstract]))) AND (("Robotics"[Mesh]) OR ((((((((((("robot"[Title/Abstract]) OR ("bot"[Title/Abstract])) OR (conversation*[Title/Abstract])) OR (chatbot[Title/Abstract])) OR (image[Title/Abstract])) OR (graph[Title/Abstract])) OR (art[Title/Abstract])) OR (music[Title/Abstract])) OR (song[Title/Abstract])) OR (video[Title/Abstract])) OR (text[Title/Abstract])))) AND (("Mental Health"[Mesh]) OR (((((((("mental health"[Title/Abstract]) OR ("mental well-being"[Title/Abstract])) OR ("mental wellbeing"[Title/Abstract])) OR (depress*[Title/Abstract])) OR (stress*[Title/Abstract])) OR (disorder[Title/Abstract])) OR (anxi*[Title/Abstract])) OR (schizophrenia [Title/Abstract])) OR (PTSD[Title/Abstract])) OR (psycholog*[Title/Abstract]))) |
| --- | --- |
| Google Scholar  (First 100 records) | (“AI” OR "Artificial Intelligence" OR “generative AI” OR “AI-generated”) AND (chatbot OR conversation* OR image OR music OR graph OR GPT OR Midjourney OR new Bing) AND (“mental health” OR depress* OR anxi* OR stress OR psycho* OR disorder OR schizophrenia OR “PTSD”) |
| Web of Science | (“AI” OR "Artificial Intelligence" OR “generative AI” OR “AI-generated”) AND (chatbot OR conversation* OR image OR music OR graph OR GPT OR Bard OR DALL-E OR Midjourney OR Bing) AND (“mental health” OR depress* OR anxiety OR stress OR psycho* OR disorder OR schizophrenia OR “PTSD”) |
| IEEE | ("All Metadata":"Artificial Intelligence" OR "All Metadata":“generative AI” OR "All Metadata":“AI-generated OR "All Metadata":"human-like”) AND ("All Metadata":chatbot OR "All Metadata":conversation* OR "All Metadata":image OR "All Metadata":music OR "All Metadata":graph OR "All Metadata":GPT OR "All Metadata":Bard OR "All Metadata":DALL-E OR "All Metadata":Midjourney OR "All Metadata":Bing) AND ("All Metadata":“mental health” OR "All Metadata":depress* OR "All Metadata":anxi* OR "All Metadata":stress OR "All Metadata":psycho* OR "All Metadata":disorder OR "All Metadata": schizophrenia OR "All Metadata": "PTSD") |
| medRxiv & bioRxiv preprint servers | (“AI” or Artificial Intelligence or “generative AI” or “AI-generated”) and (image or music or graph) and (“mental health” or depress* or anxi* or stress or psycho* or disorder or schizophrenia or “PTSD”)  (“AI” or Artificial Intelligence or “generative AI” or “AI-generated”) and (GPT or Bard or DALL-E or Midjourney or Bing) and (“mental health” or depress* or anxi* or stress or psycho* or disorder or schizophrenia or “PTSD”) |
| CNKI | (主题：精神健康 + 心理健康 + 抑郁 + 压力 + 焦虑 + 双相情感障碍 + 精神分裂症 + 创伤后应激障碍 (精确)) AND (篇关摘: 生成式AI + 生成式人工智能 + 大语言模型 + 聊天机器人 + ChatGPT + GPT + LLM + AIGC + 图像生成 + 生成式音乐 (模糊))  Disciplines: 计算机软件/精神病学/临床医学  (Subject: Mental health + Depression + stress + anxiety + bipolar disorder + Schizophrenia + Post-traumatic stress disorder (precise) Generative AI + Generative AI + large language model + Chatbot + ChatGPT + GPT + LLM + AIGC + Image generation + Generative Music (blur)  Disciplines: Computer software/Psychiatry/Clinical Medicine |
| Wanfang | (主题:(生成式人工智能 or 聊天机器人 or "ChatGPT" or "GPT" or "生成式AI" or "AIGC" or "LLM" or 图像生成 or 生成式音乐) and 主题:(精神健康 or 心理健康 or 精神分裂症 or 抑郁 or 焦虑 or 压力 or双相情感障碍 or创伤后应激障碍) not 主题:(沟槽钉板测验))  (Topic :(Generative AI or Chatbot or "ChatGPT" or "GPT" or "generative AI" or "AIGC" or "LLM" or image generation or generative music) and topic :(Mental health or mental health or Schizophrenia or depression or anxiety or stress or bipolar disorder or post-traumatic stress disorder) not subject :(Trench pegboard test)) |
