# Supplementary material for: Debate and Dilemmas Regarding Generative AI in Mental Health Care: Scoping Review
Source: Interact J Med Res. 2024 Aug 12;13:e53672. doi: 10.2196/53672 (PMC11347908; doi:10.2196/53672)
Supplement: Multimedia Appendix 2 [file ijmr_v13i1e53672_app2.docx]

Multimedia Appendix 2. Categorization of GAI models

| Type | Traditional GAI | Advanced GAI |
| --- | --- | --- |
| Definition | typically follows rule-based or retrieval systems and relies on pre-defined patterns and rules to understand and respond to user inputs [1]. | requires huge computing power including a significant amount of memory (∼1 TB) and high-end GPUs (e.g., NVIDIA V100) [2]. |
| Example of techniques used | Pattern matching, MADSlip, conceptual ontology, decision trees, predefined rules, natural language processing, Markov chain model, Dialogflow, RoBERTa [1] | Generative Pre-trained Transformer (GPT); Variational Autoencoder (VAE); Generative Adversarial Networks (GANs); Recurrent Neural Networks (RNN): Long Short-Term Memory (LSTM), Gated Recurrent Unit (GRU); Autoencoders; Transformer-based Models; Variational Neural Dialogue Models (VNDM) [3] |
