# Supplementary material for: Debate and Dilemmas Regarding Generative AI in Mental Health Care: Scoping Review
Source: Interact J Med Res. 2024 Aug 12;13:e53672. doi: 10.2196/53672 (PMC11347908; doi:10.2196/53672)
Supplement: Multimedia Appendix 3 [file ijmr_v13i1e53672_app3.docx]

**Appendix 3 - PRISMA-ScR Checklist**

| **Section** | **Item** | **Prisma-ScR checklist item** | **Reported on page #** |
| --- | --- | --- | --- |
| **Title** |  |  |  |
| **Title** | 1 | Identify the report as a scoping review. | 1 |
| **Abstract** |  |  |  |
| **Structured summary** | 2 | Provide a structured summary including, *as applicable*: background, objectives, eligibility criteria, sources of evidence, synthesis methods, results, limitations, conclusions that relate to the review question(s) and objective(s). | 1 |
| **Introduction** |  |  |  |
| **Rationale** | 3 | Describe the rationale for the review in the context of what is already known. Explain why the review question(s)/objective(s) lend themselves to a scoping review approach. | 3 |
| **Objectives** | 4 | Provide an explicit statement of the question(s) or objective(s) being addressed with reference to their key elements (e.g., population or participants, concepts and context, or other relevant framework used to conceptualize the review question(s) and/or objective(s). | 3 |
| **Methods** |  |  |  |
| **Protocol and**  **registration** | 5 | Indicate if a review protocol exists, if and where it can be accessed (e.g., web address), and, if available, provide registration information including registration number. | - |
| **Eligibility**  **criteria** | 6 | Specify characteristics of the sources of evidence (e.g., years considered, language, publication status) used as criteria for eligibility, giving rationale. | 4 |
| **Information**  **sources** | 7 | Describe all information sources (e.g., databases with dates of coverage, contact with authors to identify additional sources) in the search and date last searched. | 4 |
| **Search** | 8 | Present full electronic search strategy for at least one database, including any limits used, such that it could be repeated. | 4 |
| **Study**  **selection** | 9 | State the process for selecting sources of evidence (i.e., screening, eligibility, included in scoping review). | 5 |
| **Data**  **collection**  **process** | 10 | Describe method of data extraction from reports (e.g., piloted forms, independently, in duplicate) and any processes for obtaining and confirming data from investigators. | 5 |
| **Data items** | 11 | List and define all variables for which data were sought and any assumptions and simplifications made. | 5 |
| **Risk of bias in**  **individual**  **studies** | 12 | ***If done,*** provide a rationale for conducting a critical appraisal of included sources of evidence; describe the methods used and how this information was used in any data synthesis (if appropriate). | - |
| **Summary**  **measures** | 13 | *Not applicable for scoping reviews, since meta-analyses aren’t performed.* | - |
| **Synthesis of**  **results** | 14 | Describe the methods of handling and summarizing the data that were extracted. | 5 |
| **Risk of bias**  **across**  **studies** | 15 | *Not applicable for scoping reviews.* | - |
| **Additional analyses** | 16 | *Not applicable for scoping reviews.* | - |
| **Results** |  |  |  |
| **Study**  **selection** | 17 | Give numbers of sources of evidence screened, assessed for eligibility, and included in the review, with reasons for exclusions at each stage, ideally with a flow diagram. | 5-6 |
| **Study**  **characteristics** | 18 | For each source of evidence, present characteristics for which data were extracted and provide the citations. | 6, 7, 9-11 |
| **Risk of bias**  **within**  **studies** | 19 | ***If done,*** present data on critical appraisal of included sources of evidence (see item 12). | - |
| **Results of**  **individual**  **studies** | 20 | For each included source of evidence, present the relevant data that were collected that pertain to the review question(s) and objective(s). | 9, 10 |
| **Synthesis of**  **results** | 21 | Summarize and/or present results in relation to the review question(s) and objective(s). | 11-14 |
| **Risk of bias**  **across**  **studies** | 22 | *Not applicable for scoping reviews.* | - |
| **Additional analyses** | 23 | *Not applicable for scoping reviews.* | - |
| **Discussion** |  |  |  |
| **Summary of**  **evidence** | 24 | Summarize the main findings (including an overview of concepts, types of evidence available), link back to the review question(s) and objectives, and consider the relevance to key groups. | 14 |
| **Limitations** | 25 | Discuss the limitations of your scoping review process and of the sources of evidence that were included. | 18-19 |
| **Conclusions** | 26 | Provide a general interpretation of the results with respect to the review question(s) and objective(s), as well as potential implications and/or next steps. | 19 |
| **Funding** |  |  |  |
| **Funding** | 27 | Describe sources of funding for the scoping review and other support (e.g., individuals who provided data); as well as the role of funders for the scoping review. | 19 |

Tricco AC, Lillie E, Zarin W, et al. Prisma extension for scoping reviews (prisma-scr): Checklist and explanation. *Ann of Intern Med* 2018; **169**(7): 467-73.
