# Supplementary material for: Debate and Dilemmas Regarding Generative AI in Mental Health Care: Scoping Review
Source: Interact J Med Res. 2024 Aug 12;13:e53672. doi: 10.2196/53672 (PMC11347908; doi:10.2196/53672)
Supplement: Multimedia Appendix 4 [file ijmr_v13i1e53672_app4.docx]

Multimedia Appendix 4. List of articles included in the brief review

| **Number** | **Author** | **Year** | **Title** | **Article Type** |
| --- | --- | --- | --- | --- |
| 1 | Cameron, G., D. Cameron, G. Megaw, R. Bond, M. Mulvenna, S. O’Neill, C. Armour and M. McTear | 2017 | Towards a chatbot for digital counselling. | Conference Proceedings |
| 2 | D'Alfonso, S., O. Santesteban-Echarri, S. Rice, G. Wadley, R. Lederman, C. Miles, J. Gleeson and M. Alvarez-Jimenez | 2017 | Artificial Intelligence-Assisted Online Social Therapy for Youth Mental Health. | Journal Article |
| 3 | Ly, K. H., A.-M. Ly and G. Andersson | 2017 | A fully automated conversational agent for promoting mental well-being: A pilot RCT using mixed methods. | Journal Article |
| 4 | Miner, A. S., A. Milstein and J. T. Hancock | 2017 | Talking to machines about personal mental health problems. | Journal Article |
| 5 | Mujeeb, S., M. H. Javed and T. Arshad | 2017 | Aquabot: a diagnostic chatbot for achluophobia and autism. | Journal Article |
| 6 | Divya, S., V. Indumathi, S. Ishwarya, M. Priyasankari and S. K. Devi | 2018 | A self-diagnosis medical chatbot using artificial intelligence. | Journal Article |
| 7 | Fulmer, R., A. Joerin, B. Gentile, L. Lakerink and M. Rauws | 2018 | Using Psychological Artificial Intelligence (Tess) to Relieve Symptoms of Depression and Anxiety: Randomized Controlled Trial. | Journal Article |
| 8 | Inkster, B., S. Sarda and V. Subramanian | 2018 | An Empathy-Driven, Conversational Artificial Intelligence Agent (Wysa) for Digital Mental Well-Being: Real-World Data Evaluation Mixed-Methods Study. | Journal Article |
| 9 | Sakthivel, V., K. Srihari, S. Karthik and C. D. Anisha | 2018 | Intelligent Counselling Bot Using Ranking Algorithm in AI. | Conference Proceedings |
| 10 | Suganuma, S., D. Sakamoto and H. Shimoyama | 2018 | An Embodied Conversational Agent for Unguided Internet-Based Cognitive Behavior Therapy in Preventative Mental Health: Feasibility and Acceptability Pilot Trial. | Journal Article |
| 11 | Bao, M. H. | 2019 | Can Home Use of Speech-Enabled Artificial Intelligence Mitigate Foreign Language Anxiety - Investigation of a Concept. | Journal Article |
| 12 | Falala-Sechet, C., L. Antoine, I. Thiriez, C. Bungener and Acm | 2019 | Owlie: A Chatbot that Provides Emotional support for Coping with Psychological Difficulties. | Conference Proceedings |
| 13 | Greer, S., D. Ramo, Y.-J. Chang, M. Fu, J. Moskowitz and J. Haritatos | 2019 | Use of the chatbot vivibot to deliver positive psychology skills and promote well-being among young people after cancer treatment: randomized controlled feasibility trial. | Journal Article |
| 14 | Joerin, A., M. Rauws and M. L. Ackerman | 2019 | Psychological Artificial Intelligence Service, Tess: Delivering On-demand Support to Patients and Their Caregivers: Technical Report. | Journal Article |
| 15 | Lovejoy, C. A. | 2019 | Technology and mental health: the role of artificial intelligence. | Journal Article |
| 16 | Patel, F., R. Thakore, I. Nandwani, S. K. Bharti and Ieee | 2019 | Combating Depression in Students using an Intelligent ChatBot: A Cognitive Behavioral Therapy. | Conference Proceedings |
| 17 | Ralston, K., Y. Chen, H. Isah and F. Zulkernine | 2019 | A Voice Interactive Multilingual Student Support System using IBM Watson. | Conference Proceedings |
| 18 | Shorey, S., E. Ang, J. Yap, E. D. Ng, S. T. Lau and C. K. Chui | 2019 | A Virtual Counseling Application Using Artificial Intelligence for Communication Skills Training in Nursing Education: Development Study. | Journal Article |
| 19 | Tamilarasi, F. C. and J. Shanmugam | 2019 | Artificial Intelligence - Machine Learning based Mental Health Diagnosis Automation. | Journal Article |
| 20 | Tran, B. X., R. S. McIntyre, C. A. Latkin, H. T. Phan, G. T. Vu, H. L. T. Nguyen, K. K. Gwee, C. S. H. Ho and R. C. M. Ho | 2019 | The Current Research Landscape on the Artificial Intelligence Application in the Management of Depressive Disorders: A Bibliometric Analysis. | Journal Article |
| 21 | Vijayarani, M. and G. Balamurugan | 2019 | Chatbot in mental health care. | Journal Article |
| 22 | Adikari, A., D. de Silva, W. K. B. Ranasinghe, T. Bandaragoda, O. Alahakoon, R. Persad, N. Lawrentschuk, D. Alahakoon and D. Bolton | 2020 | Can online support groups address psychological morbidity of cancer patients? An artificial intelligence based investigation of prostate cancer trajectories. | Journal Article |
| 23 | Bharti, U., D. Bajaj, H. Batra, S. Lalit, S. Lalit and A. Gangwani | 2020 | Medbot: Conversational artificial intelligence powered chatbot for delivering tele-health after covid-19. | Conference Proceedings |
| 24 | Dosovitsky, G., B. S. Pineda, N. C. Jacobson, C. Chang and E. L. Bunge | 2020 | Artificial intelligence chatbot for depression: descriptive study of usage. | Journal Article |
| 25 | Gamble, A. | 2020 | Artificial intelligence and mobile apps for mental healthcare: a social informatics perspective. | Journal Article |
| 26 | Green, E. P., Y. H. Lai, N. Pearson, S. Rajasekharan, M. Rauws, A. Joerin, E. Kwobah, C. Musyimi, R. M. Jones, C. Bhat, A. Mulinge and E. S. Puffer | 2020 | Expanding Access to Perinatal Depression Treatment in Kenya Through Automated Psychological Support: Development and Usability Study. | Journal Article |
| 27 | Høiland, C. G., A. Følstad and A. Karahasanovic | 2020 | Hi, can I help? Exploring how to design a mental health chatbot for youths. | Journal Article |
| 28 | Lee, Y.-C., N. Yamashita and Y. Huang | 2020 | Designing a chatbot as a mediator for promoting deep self-disclosure to a real mental health professional. | Journal Article |
| 29 | Linden, B., L. Tam-Seto and H. Stuart | 2020 | Adherence of the #Here4U App - Military Version to Criteria for the Development of Rigorous Mental Health Apps. | Journal Article |
| 30 | Mitin, S. J. | 2020 | Psychological Assistant Bot Using Artificial Intelligence to Improve Individuals’ Mental Health. | Journal Article |
| 31 | Osorio, M. J. O., C. Zepeda and J. L. Carballido | 2020 | MyUBot: Towards an Artificial Intelligence Agent System Chat-bot for Well-being and Mental Health. | Conference Proceedings |
| 32 | Prakash, A. V. and S. Das | 2020 | Intelligent Conversational Agents in Mental Healthcare Services: A Thematic Analysis of User Perceptions. | Journal Article |
| 33 | Ta, V., C. Griffith, C. Boatfield, X. Y. Wang, M. Civitello, H. Bader, E. DeCero and A. Loggarakis | 2020 | User Experiences of Social Support From Companion Chatbots in Everyday Contexts: Thematic Analysis. | Journal Article |
| 34 | Thomas, N., A. Perumalla, S. Rao, V. Thangaraj, K. S. Ravi, S. Geethanath, H. Kim and G. Srinivasan | 2020 | Fully Automated End-to-End Neuroimaging Workflow for Mental Health Screening. | Conference Proceedings |
| 35 | Williams, D., V. J. Hodge and C. Y. Wu | 2020 | On the use of AI for Generation of Functional Music to Improve Mental Health. | Journal Article |
| 36 | Beilharz, F., S. Sukunesan, S. L. Rossell, J. Kulkarni and G. Sharp | 2021 | Development of a Positive Body Image Chatbot (KIT) With Young People and Parents/Carers: Qualitative Focus Group Study. | Journal Article |
| 37 | Chung, K., H. Y. Cho and J. Y. Park | 2021 | A chatbot for perinatal women’s and partners’ obstetric and mental health care: Development and usability evaluation study. | Journal Article |
| 38 | Danieli, M., T. Ciulli, S. M. Mousavi and G. Riccardi | 2021 | A Conversational Artificial Intelligence Agent for a Mental Health Care App: Evaluation Study of Its Participatory Design. | Journal Article |
| 39 | Denecke, K., A. Abd-Alrazaq and M. Househ | 2021 | Artificial intelligence for chatbots in mental health: opportunities and challenges. | Journal Article |
| 40 | Grove, C. | 2021 | Co-developing a Mental Health and Wellbeing Chatbot With and for Young People. | Journal Article |
| 41 | Gunawan, T. S., A. B. F. Babiker, N. Ismail and M. R. Effendi | 2021 | Development of Intelligent Telegram Chatbot Using Natural Language Processing. | Conference Proceedings |
| 42 | Hungerbuehler, I., K. Daley, K. Cavanagh, H. Garcia Claro and M. Kapps | 2021 | Chatbot-based assessment of employees’ mental health: Design process and pilot implementation. | Journal Article |
| 43 | Jameel, U., A. Anwar and H. Khan | 2021 | Doctor recommendation chatbot: A research study: Doctor recommendation chatbot. | Journal Article |
| 44 | Kaywan, P., K. Ahmed, Y. Miao, A. Ibaida and B. Gu | 2021 | DEPRA: An Early Depression Detection Analysis Chatbot. | Conference Proceedings |
| 45 | Klos, M. C., M. Escoredo, A. Joerin, V. N. Lemos, M. Rauws and E. L. Bunge | 2021 | Artificial intelligence–based chatbot for anxiety and depression in university students: pilot randomized controlled trial. | Journal Article |
| 46 | Lee, J., E. Sezgin, J. Bridge, S. Lin and S. Yang | 2021 | Clinical Advice by Voice Assistants on Postpartum Depression: Cross-Sectional Investigation Using Apple Siri, Amazon Alexa, Google Assistant, and Microsoft Cortana. | Journal Article |
| 47 | Madzin, H., N. D. K. Kamarol and S. K. Ali | 2021 | Re: Feel- Muslim Self-Help Mobile Application with Asma'ul Husna. | Conference Proceedings |
| 48 | Mauriello, M. L., N. Tantivasadakarn, M. A. Mora-Mendoza, E. T. Lincoln, G. Hon, P. Nowruzi, D. Simon, L. Hansen, N. H. Goenawan, J. Kim, N. Gowda, D. Jurafsky and P. E. Paredes | 2021 | A Suite of Mobile Conversational Agents for Daily Stress Management (Popbots): Mixed Methods Exploratory Study. | Journal Article |
| 49 | Meng, J. B. and Y. Dai | 2021 | Emotional Support from AI Chatbots: Should a Supportive Partner Self-Disclose or Not? | Journal Article |
| 50 | Patole, A., V. Dumbre, R. Kesharwani and H. Khanuja | 2021 | Mental Health Chatbot (Psykh). | Journal Article |
| 51 | Potts, C., E. Ennis, R. Bond, M. Mulvenna, M. F. McTear, K. Boyd, T. Broderick, M. Malcolm, L. Kuosmanen and H. Nieminen | 2021 | Chatbots to Support Mental Wellbeing of People Living in Rural Areas: Can User Groups Contribute to Co-design? | Journal Article |
| 52 | Prochaska, J. J., E. A. Vogel, A. Chieng, M. Kendra, M. Baiocchi, S. Pajarito and A. Robinson | 2021 | A therapeutic relational agent for reducing problematic substance use (Woebot): development and usability study. | Journal Article |
| 53 | Romanovskyi, O., N. Pidbutska and A. Knysh | 2021 | Elomia Chatbot: the Effectiveness of Artificial Intelligence in the Fight for Mental Health. | Conference Proceedings |
| 54 | Sturgill, R., M. Martinasek, T. Schmidt and R. Goyal | 2021 | A Novel Artificial Intelligence-Powered Emotional Intelligence and Mindfulness App (Ajivar) for the College Student Population During the COVID-19 Pandemic: Quantitative Questionnaire Study. | Journal Article |
| 55 | Wibhowo, C. and R. Sanjaya | 2021 | Virtual assistant to suicide prevention in individuals with borderline personality disorder. | Conference Proceedings |
| 56 | Adikari, A., D. de Silva, H. Moraliyage, D. Alahakoon, J. H. Wong, M. Gancarz, S. Chackochan, B. Park, R. Heo and Y. Leung | 2022 | Empathic conversational agents for real-time monitoring and co-facilitation of patient-centered healthcare. | Journal Article |
| 57 | Ahmad, R., D. Siemon, U. Gnewuch and S. Robra-Bissantz | 2022 | Designing Personality-Adaptive Conversational Agents for Mental Health Care. | Journal Article |
| 58 | Anastasiia, M., T. Korotyeva and Ieee | 2022 | A chatbot of a person's emotional state using a neural network. | Conference Proceedings |
| 59 | Bishop, D. | 2022 | A friend within your phone: The benefits and harms of social chatbot Replika. |  |
| 60 | Chan, W. W., E. E. Fitzsimmons-Craft, A. C. Smith, M.-L. Firebaugh, L. A. Fowler, B. DePietro, N. Topooco, D. E. Wilfley, C. B. Taylor and N. C. Jacobson | 2022 | The challenges in designing a prevention chatbot for eating disorders: observational study. | Journal Article |
| 61 | Danieli, M., T. Ciulli, S. M. Mousavi, G. Silvestri, S. Barbato, L. Di Natale and G. Riccardi | 2022 | Assessing the Impact of Conversational Artificial Intelligence in the Treatment of Stress and Anxiety in Aging Adults: Randomized Controlled Trial. | Journal Article |
| 62 | He, Y., L. Yang, B. W. BE, S. Z. BE, C. Qian and T. T. BE | 2022 | Mental health chatbot for young adults with depressive symptoms: a single-blind, three-arm, randomized controlled trial. | Journal Article |
| 63 | He, Y., L. Yang, X. Zhu, B. Wu, S. Zhang, C. Qian and T. Tian | 2022 | Mental health chatbot for young adults with depressive symptoms during the COVID-19 pandemic: single-blind, three-arm randomized controlled trial. | Journal Article |
| 64 | Ismail, I. E., D. Y. Liliana and A. R. Zain | 2022 | Evaluation of EmoHealth Application Using USE Questionnaire. | Conference Proceedings |
| 65 | Laestadius, L., A. Bishop, M. Gonzalez, D. Illencik and C. Campos-Castillo | 2022 | Too human and not human enough: A grounded theory analysis of mental health harms from emotional dependence on the social chatbot Replika. | Journal Article |
| 66 | Lee, J., D. Lee and J. G. Lee | 2022 | Influence of Rapport and Social Presence with an AI Psychotherapy Chatbot on Users' Self-Disclosure. | Journal Article |
| 67 | Liu, H., H. M. Peng, X. Y. Song, C. Z. Xu and M. Zhang | 2022 | Using AI chatbots to provide self-help depression interventions for university students: A randomized trial of effectiveness. | Journal Article |
| 68 | Liu, Y. C., S. Xia, J. P. Nie, P. Wei, Z. Shu, J. A. Chang and X. F. Jiang | 2022 | aiMSE: Toward an AI-Based Online Mental Status Examination. | Journal Article |
| 69 | Lokala, U., A. Srivastava, T. G. Dastidar, T. Chakraborty, M. S. Akhtar, M. Panahiazar and A. Sheth | 2022 | A computational approach to understand mental health from reddit: knowledge-aware multitask learning framework. | Conference Proceedings |
| 70 | Mehta, A., S. Virkar, J. Khatri, R. Thakur and A. Dalvi | 2022 | Artificial Intelligence Powered Chatbot for Mental Healthcare based on Sentiment Analysis. | Conference Proceedings |
| 71 | Moilanen, J., A. Visuri, S. A. Suryanarayana, A. Alorwu, K. Yatani and S. Hosio | 2022 | Measuring the Effect of Mental Health Chatbot Personality on User Engagement. | Conference Proceedings |
| 72 | Natsheh, E. and M. Jabed | 2022 | Implementing artificial intelligence-based COVID-19 chatbot in the kingdom of bahrain. | Journal Article |
| 73 | Nayar, A. M., Z. Attar, S. Kachwala, T. Biswas and S. K. Wagh | 2022 | Dost-Mental Health Assistant Chatbot. | Conference Proceedings |
| 74 | Nelekar, S., A. Abdulrahman, M. Gupta and D. Richards | 2022 | Effectiveness of embodied conversational agents for managing academic stress at an Indian University (ARU) during COVID-19. | Journal Article |
| 75 | Ogawa, M., G. Oyama, K. Morito, M. Kobayashi, Y. Yamada, K. Shinkawa, H. Kamo, T. Hatano and N. Hattori | 2022 | Can AI make people happy? The effect of AI-based chatbot on smile and speech in Parkinson's disease. | Journal Article |
| 76 | Ouerhani, N., A. Maalel, H. Ben Ghezala and Ieee | 2022 | Towards a smart pervasive conversational agent for COVID-19 psychological assistance based on NLP. | Conference Proceedings |
| 77 | Pandey, S., S. Sharma and S. Wazir | 2022 | Mental healthcare chatbot based on natural language processing and deep learning approaches: ted the therapist. | Journal Article |
| 78 | Rathnayaka, P., N. Mills, D. Burnett, D. De Silva, D. Alahakoon and R. Gray | 2022 | A Mental Health Chatbot with Cognitive Skills for Personalised Behavioural Activation and Remote Health Monitoring. | Journal Article |
| 79 | Siemon, D., R. Ahmad, H. Harms and T. de Vreede | 2022 | Requirements and Solution Approaches to Personality-Adaptive Conversational Agents in Mental Health Care. | Journal Article |
| 80 | Sulaiman, S., M. Mansor, R. A. Wahid and N. A. A. N. Azhar | 2022 | Anxiety Assistance Mobile Apps Chatbot Using Cognitive Behavioural Therapy. | Journal Article |
| 81 | Ta-Johnson, V. P., C. Boatfield, X. Y. Wang, E. DeCero, I. C. Krupica, S. Rasof, A. Motzer and W. M. Pedryc | 2022 | Assessing the Topics and Motivating Factors Behind Human-Social Chatbot Interactions: Thematic Analysis of User Experiences. | Journal Article |
| 82 | Thakur, S., D. Rastogi and L. Singh | 2022 | MOODY: A Natural Language Processing-Based Chatbot for Mental Health Care. | Book Section |
| 83 | Zhu, Y., R. Wang and C. Pu | 2022 | I am chatbot, your virtual mental health adviser. What drives citizens’ satisfaction and continuance intention toward mental health chatbots during the COVID-19 pandemic? An empirical study in China. | Journal Article |
| 84 | 王海舟 | 2022 | 基于改進的BERT-TextCNN和知識圖譜的青少年心理預警研究 (Artificial intelligent depression based on sleep cardiogram data). | Thesis |
| 85 | 肖碧波, 唐健豪, 曾加玉, 王肖文 and 魏晓滨 | 2022 | 基于睡眠心电数据的人工智能抑郁评估系统 (Artificial intelligent depression based on sleep cardiogram data). | Journal Article |
| 86 | 張慧 | 2022 | 基于文本預訓練模型的抑郁傾向檢測方法研究 (A study of depression tendency detection based on text pretraining model). | Thesis |
| 87 | Apablaza, J. and S. Cano | 2023 | Model to Design Affective Conversational Interfaces to Support the Mental Health. | Conference Proceedings |
| 88 | Booth, F., C. Potts, R. Bond, M. Mulvenna, C. Kostenius, I. Dhanapala, A. Vakaloudis, B. Cahill, L. Kuosmanen and E. Ennis | 2023 | A Mental Health and Well-Being Chatbot: User Event Log Analysis. | Journal Article |
| 89 | Ghoshal, N., V. Bhartia, B. Tripathy and A. Tripathy | 2023 | Chatbot for Mental Health Diagnosis Using NLP and Deep Learning. | Book Section |
| 90 | Iglesias, M., C. Sinha, R. Vempati, S. E. Grace, M. Roy, W. C. Chapman and M. L. Rinaldi | 2023 | Evaluating a Digital Mental Health Intervention (Wysa) for Workers' Compensation Claimants Pilot Feasibility Study. | Journal Article |
| 91 | Inkster, B., M. Kadaba and V. Subramanian | 2023 | Understanding the impact of an AI-enabled conversational agent mobile app on users' mental health and wellbeing with a self-reported maternal event: a mixed method real-world data mHealth study. | Journal Article |
| 92 | Malhotra, S. | 2023 | Mental Health Apps: A New Field in Community Mental Health Care. | Journal Article |
| 93 | Park, D. Y. and H. Kim | 2023 | Determinants of Intentions to Use Digital Mental Healthcare Content among University Students, Faculty, and Staff: Motivation, Perceived Usefulness, Perceived Ease of Use, and Parasocial Interaction with AI Chatbot. | Journal Article |
| 94 | Park, G., S. Y. Lee and J. Y. Chung | 2023 | Do Anthropomorphic Chatbots Increase Counseling Satisfaction and Reuse Intention? The Moderated Mediation of Social Rapport and Social Anxiety. | Journal Article |
| 95 | Park, I., S. Lee and D. Lee | 2023 | Virtual Audience Providing AI-Generated Emotional Reactions to Enhance Self-Disclosure in Self-Introduction. | Journal Article |
| 96 | Podina, I. R. and D. Caculidis-Tudor | 2023 | Increasing Well-Being and Mental Health Through Cutting-Edge Technology and Artificial Intelligence. | Book Section |
| 97 | Shahsavar, Y. and A. Choudhury | 2023 | User Intentions to Use ChatGPT for Self-Diagnosis and Health-Related Purposes: Cross-sectional Survey Study. | Journal Article |
| 98 | Sinha, C., S. Meheli and M. Kadaba | 2023 | Understanding Digital Mental Health Needs and Usage With an Artificial Intelligence-Led Mental Health App (Wysa) During the COVID-19 Pandemic: Retrospective Analysis. | Journal Article |
| 99 | Suharwardy, S., M. Ramachandran, S. A. Leonard, A. Gunaseelan, D. J. Lyell, A. Darcy, A. Robinson and A. Judy | 2023 | Feasibility and impact of a mental health chatbot on postpartum mental health: a randomized controlled trial. | Journal Article |
| 100 | Wilson, R. L., O. Higgins, J. Atem, A. E. Donaldson, F. A. Gildberg, M. Hooper, M. Hopwood, S. Rosado, B. Solomon and K. Ward | 2023 | Artificial intelligence: An eye cast towards the mental health nursing horizon. | Journal Article |
